# Miscellany

**Published:** 1887-11

**Authors:** 


### Miscellany.

Preventive Inoculations for Scarlet Fever.—The day is probably not far distant when scarlet fever, at present the greatest scourge of childhood, will be as effectually prevented by inoculation as small-pox is by vaccination. Stickler of Paris observed that horses are often affected by a disease quite similar to scarlet fever, and, indeed, it is sometimes known to veterinarians by that name. He inserted about six drops of the nasal discharge of the diseased horse under the skin of four rabbits and dog, and, within twenty-four hours, an exanthema resembling scarlatina appeared, which was followed in four days by desquamation. The associated symptoms were: fever, anorexia, redness of the nasal mucus membrane with abundant secretion, and enlargement of the lymphatic glands. The animals recovered in eight days, and the inoculation of scarlatinal blood gave negative results.

Stickler then inoculated twelve children with the mucus of the horse. In every case, a punctiform eruption appeared within twenty-four hours, and was accompanied by fever, and enlargement of the glands. The eruption lasted six days and terminated in desquamation. After recovery, these children were inoculated with scarlatinal blood with absolutely negative results.—*Gazette Med. de Paris.*

---

THE STENOCARPINE SENSATION—AN ATTEMPT TO IMPOSE UPON THE MEDICAL PROFESSION.—Our readers are, doubtless, familiar with the reports of Drs. J. H. Claiborne, Hermann Knapp and Edward Jackson, concerning the so-called new local anæsthetic, gleditschine or stenocarpine, which was published in the New York *Medical Record* July 30th, August 13th and October 1st, and Philadelphia *Medical News*, September 3d, in which gleditschine was claimed to possess remarkable anæsthetic and mydriatic properties. It will, therefore, be of interest to them to learn that Messrs. Parke, Davis & Co. announce that an investigation at their laboratory, of a solution purporting to be a two per cent. solution of gleditschine or stenocarpine, which was supplied by Messrs. Lehn & Fink, of New York, has developed the fact that this solution, with which the experiments thus far recorded have been made, contains six per cent. of cocaine and a sulphate of a salt which further experiment is likely to prove to be atropia. F. A. Thompson, Ph. C., also reports, after careful experiment with the leaves of gleditschia triacanthos, from which gleditschine or stenocarpine is claimed to have been derived, that they contain only an infinitesimal percentage of an amorphous alkaloid devoid of anæsthetic or mydriatic properties. In the light of these facts, it seems probable that the stenocarpine sensation should be classed with the hopeine fraud of malodorous memory, and that the physicians who have already published reports regarding gleditschine or stenocarpine have been the victims of a clever hoax.

A NEW EDITION OF GRAY'S.—Any marked change in a text-book so universally in use as Gray's "Anatomy," becomes a matter of no inconsiderable interest to the profession, and the announcement of a new and improved edition from the press of Lea Brothers & Co. will be received with interest. While thoroughly revised, the new edition possesses a marked feature in the use of colors in illustration wherever they can be of service in enabling the eye to more readily follow the details of anatomical construction. The care requisite for the proper printing of this colored edition has involved a delay, but the book will be issued early in November. Notwithstanding the unavoidable increase in size, the issue in black has been retained at its old price—$6.00 in cloth, or $7.00 in full leather, while for that in color but $1.25 extra will be charged.

---

"DOCTOR, what do you think of this new treatment of consumption by injecting carbonic acid into the rectum?"

"Well, it certainly distracts the mind of the patient for awhile, and may do good by its moral effect by making him think of his latter end; he becomes, as it were, conscious of his rectum, as the schoolboy rendered *mens conscia recti.* He may even 'bacilli' enough to cherish the hope that he is getting cured, and is taking a short cut to health over this *pons asinorum*, but I regret to say that I think he will find that the road will end as it began—in gas."—*Med. World.*

---

LANCEREAUX has published reports of cases tending to prove that cirrhosis of the liver is curable by the adoption of a strict milk diet, and the administration of iodide of potassium. He has succeeded comparatively well in the treatment of atrophic cases, but, where there is hypertrophy, the prognosis is not encouraging. The duration of the treatment varies with the severity and chronicity of the disease. Generally, at the end of fifteen days, there is marked improvement, but the treatment must be continued at least for a year.—*Jour. de Med. de Paris.*

---

SEGRETAS recommends faradisation of the gall bladder for the cure of catarrhal jaundice. This is done by passing one electrode into the rectum and placing the positive electrode over the gall bladder. The current is to be passed about ten minutes each day.

A convenient Formula for the Treatment of Diabetes by Lithium in Pill Form.—Vigier recommends the following as more convenient than Martineau's arsenical liquid containing lithium:

℞—Lithii carbonat. . . . . . . . . . . . . . . gr. iss.
Sodii arseniat. . . . . . . . . . . . . . . . gr. 1-25.
Ext. gentian . . . . . . . . . . . . . . . gr. ¾.
For each pill.

To be taken morning and night, and continued until sugar has disappeared from the urine.

---

Diphtheria has assumed an epidemic form in some portions of Buffalo. The Board of Health should take some active measures to enforce isolation in all cases of infectious disease. In Michigan and in the neighboring city of Rochester, it is customary to placard houses in which there are patients suffering from infectious diseases. Perhaps, the adoption of this method in Buffalo would tend to arrest the spread of diphtheria and scarlet fever.

---

The fifteenth annual meeting of the American Public Health Association will be held in Memphis, Tenn., November 8th, 9th, 10th and 11th. The following are the topics for discussion: First—"The Pollution of Water-Supplies"; second—"The Disposal of Refuse Matter of Cities"; third—"The Disposal of Refuse Matter of Villages, Summer Resorts and Isolated Tenements"; fourth—"Animal Diseases Dangerous to Man."

---

The Western Pennsylvania Medical College at Pittsburgh began its second annual regular course on Tuesday, September 27th, with a class of near 100. The introductory address was delivered by the Secretary of the Faculty, Prof. W. J. Asdale. This college requires an entrance examination, and provides a three years' graded course.

---

Newest Treatment for Whooping Cough.—Resorcin and cocaine sprays—two to eight grains cocaine to the ounce of water; resorcin, two to four grains to the ounce of water, the cocaine spray being used every two hours, the resorcin every three hours.

---

Dr. Lieberkuhn, Professor of Anatomy at Marburg, died recently at the age of seventy-five.

THERE have been eighteen deaths from Asiatic cholera in New York harbor during the past month. The republic of Mexico has declared cholera epidemic (!) in New York, and will enforce a rigid quarantine against all American vessels.

---

WHEN vaginal pruritus is found in an elderly patient, do not fail to examine the urine for sugar. Vain will be the use of antipruritics until the diet of the patient is regulated and other treatment instituted.

---

A BELGIAN oculist, Ignace Peczeli, claims that he can diagnosticate all diseases and determine their etiology by examining the eyes. This reminds us of the American specialist who always examined the heart through the vaginal speculum.

---

M. FREUND has discovered that the coagulation of blood may be perverted by placing it in a vessel, the sides of which are covered with vaseline, and covering it with a film of oil.

---

PROF. ALONZO CLARK, M. D., LL.D., for many years connected with the College of Physicians and Surgeons, died in New York in September. He was in his eighty-first year.

---

TO GIVE QUININE.—Quinine given with dilute hydrobromic acid is more pleasantly received by many patients, its cerebral effects being much mitigated.

---

AN INDUCEMENT.—An Australian doctor advertises to pay one-half the funeral expenses in cases in which he is not successful.

---

ACCORDING to a writer in *Science*, every fifth person in the Texas grazing region is affected with the beef tape-worm.

---

A FRENCH writer has recently published, in a medical journal, an article on the "Physiology of Kissing."

